# The Effects of Model Insoluble Copper Compounds in a Sedimentary Environment on Denitrifying Anaerobic Methane Oxidation (DAMO) Enrichment

**DOI:** 10.3390/microorganisms12112259

**Published:** 2024-11-07

**Authors:** Longfei Xia, Yong Wang, Peiru Yao, Hodon Ryu, Zhengzhong Dong, Chen Tan, Shihai Deng, Hongjian Liao, Yaohuan Gao

**Affiliations:** 1 School of Human Settlements and Civil Engineering, Xi’an Jiaotong University, 19-3027 iHarbour Campus, Xi’an 710115, China; summer321xia@foxmail.com (L.X.); wangyong123@stu.xjtu.edu.cn (Y.W.); ypr0229@stu.xjtu.edu.cn (P.Y.); dzz_399@stu.xjtu.edu.cn (Z.D.); 202321838@stumail.nwu.edu.cn (C.T.); shihai.deng@xjtu.edu.cn (S.D.); hjliao@mail.xjtu.edu.cn (H.L.); 2 Shaanxi Provincial Land Engineering Construction Group, Xi’an 710075, China; 3 Institute of Global Environmental Change, Xi’an Jiaotong University, 19-3027 iHarbour Campus, Xi’an 710115, China; 4 United States Environmental Protection Agency, Office of Research and Development, Cincinnati, OH 45268, USA; ryu.hodon@epa.gov

**Keywords:** DAMO, *Candidatus* Methylomirabilis spp., *Candidatus* Methanoperedens spp., malachite, covellite

## Abstract

The contribution of denitrifying anaerobic methane oxidation (DAMO) as a methane sink across different habitats, especially those affected by anthropogenic activities, remains unclear. Mining and industrial and domestic use of metals/metal-containing compounds can all cause metal contamination in freshwater ecosystems. Precipitation of metal ions often limits their toxicity to local microorganisms, yet microbial activity may also cause the redissolution of various precipitates. In contrast to most other studies that apply soluble metal compounds, this study investigated the responses of enriched DAMO culture to model insoluble copper compounds, malachite and covellite, in simulated sedimentary environments. Copper ≤ 0.22 µm from covellite appeared to cause immediate inhibition in 10 h. Long-term tests (54 days) showed that apparent methane consumption was less impacted by various levels of malachite and covellite than soluble copper. However, the medium-/high-level malachite and covellite caused a 46.6–77.4% decline in denitrification and also induced significant death of the representative DAMO microorganisms. Some enriched species, such as *Methylobacter tundripaludum*, may have conducted DAMO or they may have oxidized methane aerobically using oxygen released by DAMO bacteria. Quantitative polymerase chain reaction analysis suggests that *Candidatus* Methanoperedens spp. were less affected by covellite as compared to malachite while *Candidatus* Methylomirabilis spp. responded similarly to the two compounds. Under the stress induced by copper, DAMO archaea, *Planctomycetes* spp. or *Phenylobacterium* spp. synthesized PHA/PHB-like compounds, rendering incomplete methane oxidation. Overall, the findings suggest that while DAMO activity may persist in ecosystems previously exposed to copper pollution, long-term methane abatement capability may be impaired due to a shift of the microbial community or the inhibition of representative DAMO microorganisms.

## 1. Introduction

Denitrifying anaerobic methane oxidation (DAMO) is an important methane sink in freshwater wetlands, terrestrial, and even coastal ecosystems where robust methanogenesis occurs due to abundant organic carbon input [1,2,3,4,5,6,7,8]. The DAMO process is currently known to be conducted by two groups of microorganisms, namely DAMO bacteria, represented by *Candidatus* Methylomirabilis oxyfera, and DAMO archaea, represented by *Candidatus* Methanoperedens nitroreducens [9,10,11]. The exact contribution of DAMO to the global methane sink is currently unclear due to the shortage of knowledge regarding DAMO activity across a wide variety of habitats [12,13]. Compared with methanogenesis or aerobic methanotrophy, progress in the mechanistic studies of DAMO has only been made recently, and the interaction between DAMO and their surroundings is just beginning to be investigated [14,15,16,17].

With a growing global population and increased urbanization, many freshwater and wetland ecosystems in inland and coastal areas are subject to heavy metal pollution [18]. Additionally, industrial activities such as metal mining, active and inactive, also contribute to the metal contamination of rivers and their associated floodplains globally [19]. Microorganisms in the above-mentioned ecosystems are inevitably influenced by various contaminants that we discharge into the environment. In environmental niches such as the freshwater sedimentary ecosystems, where DAMO microorganisms are often active, heavy metal ions usually precipitate with sulfide within a few days [20]. Although precipitation as carbonate or sulfide salts may limit metal ion toxicity to microorganisms, those heavy metals that are essential to microbial metabolism can be released via microbiological weathering of solids deposited in sediments [21,22]. Copper is one of the essential elements for microorganisms. For instance, DAMO bacteria express a membrane-bound Cu-containing enzyme (particulate methane monooxygenase, pMMO) that catalyzes the first step of methane oxidation, similarly to aerobic methanotrophs. Moreover, key denitrification enzymes, such as nitrite reductase, require copper as a cofactor [23]. Therefore, on the one hand, the availability of essential metals in methanotrophy, including Fe, Cu, Co, and Ni, may be limited due to sulfide scavenging; on the other hand, the dissolution and transformation of the seemingly inert sulfide or carbonate compounds may exert still unknown influence on DAMO activity. Kulczycki et al. examined the effect of solid phase copper on methane oxidation by *Methylosinus trichosporium* OB3b, a model aerobic methanotrophic bacterium, and found that a solid copper concentration of 80–200 µg/g led to the highest methane oxidation rates [24]. Until now, relevant studies concerning DAMO are still missing.

Available information on the effect of heavy metals (e.g., Fe, Cu, Sb, As) on DAMO is usually based on continuously mixed membrane bioreactors [25,26,27,28,29,30,31], and soluble compounds are usually applied. The conclusions from these studies cannot be extrapolated to natural habitats of DAMO microorganisms, where solid forms of heavy metals are more prevalent.

The objective of this study was to examine the response of DAMO microorganisms and the methane oxidation-based denitrification process to two model insoluble copper phases [20,32], malachite and covellite, in a simulated and simplified freshwater ecosystem. Enriched culture was used to better demonstrate the responses from DAMO microorganisms. To the best of our knowledge, this study represents a first attempt to examine the DAMO activity in a copper-contaminated freshwater sedimentary environment.

## 2. Materials and Methods

### 2.1. Inoculum and the Enrichment of DAMO Microorganisms

The inoculum was collected from the bank of the River Wei near our campus (GPS Coordinates 108.6706473°, 34.2831757°), known for its low background heavy metal levels [33]. Approximately 30 g of sediment from the top 20 cm was transferred to glass bottles (working volume of 315 mL), and 150 mL of medium [34,35] was added, leaving 165 mL of headspace. Nitrate and nitrite were both supplied to enrich DAMO microorganisms (0.5 g KNO_3_/L and 0.0345 g NaNO_2_/L initially) [35]. The incubation lasted for approximately 500 days, and more details of the operation are available in the Supplementary Materials. All tests shown below were based on the enrichment.

### 2.2. The Tests for Copper Influence and the Associated Copper Compound Synthesis

Details for the synthesis of basic copper carbonate (Cu_2_[OH]_2_CO_3_, simplified hereafter as CuCO_3_) and copper sulfide (CuS) can be found in the Supplementary Materials. After being synthesized, the compounds were thoroughly rinsed with ultrapure water and dried (105 °C) before use. The toxicity of copper on the DAMO enrichment was examined by a short-term test and a long-term test.

The short-term test lasted for 10 h, using active volatile suspended solids (AVSS) as an indicator. Only the low level of copper dosage (1 mg Cu/L) was investigated in this test. Samples were collected at the beginning, and 2 h, 6 h, and 10 h after copper addition.

The long-term test lasted for 54 days. Briefly, 100 mL bottles with a solid and liquid volume of approximately 55 mL (6 g sedimentary slurry from the enrichment phase with 50 mL liquid; average solid content of the slurry was 29%) and a 66.5 mL headspace were used. The solid copper was suspended in the base medium for a 100 mg Cu/L stock solution and distributed to low-, medium-, and high-dosage groups for a final concentration of 1 mg Cu/L, 50 mg Cu/L, and 80 mg Cu/L via mixing with the medium mentioned in Section 2.1. A separate group was dosed with only soluble copper (CuCl_2_) at the same copper levels. To better reveal the effect of copper, a group without additional copper dosage was included and is named “regular” or “normal” hereafter (i.e., only the base medium and the sedimentary slurry, as the biotic controls). Additionally, each of the aforementioned groups was paired with corresponding abiotic controls (autoclaved, 30 min at 121 °C), named “abiotic” hereafter. Since the abiotic controls yielded no methane or nitrate/nitrite consumption except for the fluctuation introduced by instrumental analysis, they were not reported separately in the Results Section. Each test condition has three independent incubations.

The sealed bottles underwent at least three cycles of vacuuming and nitrogen filling. The headspace pressure was left at 1 atm, and then 7 mL of ultrapure methane was injected into each bottle with a gas-tight syringe. The headspace methane was sampled (50 µL, SGE 005250, Trajan Scientific and Medical, Ringwood, Australia) for analysis on the second day and then every three days thereafter.

In the end, the bottles were opened in a glove box and the liquid was sampled for pH, NO_3_^−^/NO_2_^−^, SO_4_^2−^, and soluble copper analysis. The slurry was used for staining, adenosine triphosphate (ATP), total copper, metal sequential extraction (Supplementary Materials), quantitative polymerase chain reaction (qPCR), and metagenomic analysis. More details of sampling, analysis, and computation can be found in the Supplementary Materials.

### 2.3. Slurry Sampling, Staining, DNA Extraction, Library Preparation and Metagenome Sequencing, and Bioinformatics

For Nile Red (Heowns, Tianjin, China) staining, slurry from each bottle was transferred to a centrifuge tube and underwent hand shaking, ultrasonication (200 W, 5 °C, 3 min) to separate microorganisms from the sediment [36], and centrifugation (100× *g* force, 5 °C, 5 min) to collect the cells [37]. The extracted pellets were then resuspended in 1 × PBS for staining. Nile Red was first dissolved in ethanol (1 mg/mL) and then diluted 10× with ethanol and mixed with cells (50 µL Nile Red in 2.45 mL suspension, 30 min contact). Cells after staining were then centrifuged (6000× *g*, 5 °C, 5 min), resuspended with ultrapure water, and fixed on glass slides. A Nikon eclipse Ti-E epifluorescence microscope (Nikon Corporate, Tokyo, Japan) was used for observation.

For DNA extraction, the slurry from each tested bottle, approximately 5 g with approximately 29% solids, was used (PowerSoil DNA Isolation Kit, Mo Bio Laboratories Inc., Carlsbad, 92010 CA, USA) following the manufacturer’s instructions. DNA integrity and purity were monitored on 1% agarose gels. A NanoDrop ND2000 (Thermo Scientific, Waltham, 02451 MA, USA) spectrophotometer and a Qubit 3.0 (Thermo Fisher Scientific, Waltham, 02451 MA, USA) were used to check the DNA purity and concentration, respectively, before Illumina sequencing. Sequencing libraries were generated using ALFA-SEQ DNA Library Prep Kit following manufacturer’s recommendations, and index codes were added. The library quality was assessed using the Qubit 4.0 Fluorometer (Thermo Fisher Scientific, Waltham, 02451 MA, USA) and the Qsep400 High-Throughput Nucleic Acid Protein Analysis System (Houze Biological Technology Co., Hangzhou, China). Finally, the library was sequenced on an Illumina NovaSeq 6000 platform, and 150 bp paired-end reads were generated. The sequencing was performed by Magigene Biotechnology Co., Ltd. (Guangzhou, China).

For the sequencing data processing, the raw data were processed using Trimmomatic (v.0.36): http://www.usadellab.org/cms/index.php?page=trimmomatic (accessed on 20 March 2023) to remove adapter sequences, and the quality filter was carried out using fastq_quality_filter from the FASTX toolkit with default settings. The processed sequencing data, called Clean Data hereafter, were used for subsequent analysis. More details can be found in Supplementary Materials. All the raw sequence data were deposited in the NCBI database, and the BioProject accession number is PRJNA1010380.

### 2.4. Quantitative Polymerase Chain Reaction (qPCR) Analysis

Quantitative PCR was performed on an Applied Biosystems™ QuantStudio™ 5 realtime PCR instrument, Waltham, MA, USA. The abundance of DAMO bacterial *pmoA* and DAMO archaeal *mcrA* genes was quantified with the primer pair of cmo182/cmo568 [38] and McrA159F/McrA345R [39], respectively (details of primers and reaction processes can be found in Table S1). The primers were synthesized by Huayu Gene (Wuhan, China). The abundances of total bacteria and total archaea (16S rRNA gene) were quantified with the primer pairs of 341F/518R and Arch967F/Arch1060R [40], respectively. Each reaction mixture (30 μL) consisted of 15 μL 2×ChamQ SYBR qPCR Master Mix (Vazyme, Nanjing, China), 1 μL of each primer (10 μM), and 1 μL of DNA template and 12 μL of ddH_2_O. Negative controls were run with sterilized distilled water as the template instead of the DNA sample. The qPCR was conducted in triplicate in 96-well optical plates. Standard curves were obtained with serial dilutions of plasmid DNA containing the target genes. Amplification efficiencies of 90–110% with correlation coefficients above 0.98 were adopted.

### 2.5. Instrumental Analysis

Methane in the headspace was monitored with a gas chromatograph (GC) equipped with a thermal conductivity detector (TCD) and a flame ionization detector (FID) (Agilent 8860, Santa Clara, 95051 CA, USA), following the methods published elsewhere [41]. Dissolved methane was computed based on headspace pressure (measured with a precision pressure transducer) and Henry’s Law (Supplementary Materials). Nitrate and nitrite were quantified with an ion chromatograph (Dionex Aquion, Chelmsford, 01824 MA, USA), following the method published elsewhere [42]. After being synthesized and thoroughly dried, the solid copper compounds were characterized using X-ray diffraction (XRD) and scanning electron microscope (SEM) for identification and basic physical properties (Supplementary Materials). ATP was quantified with the QG21W-50C kit and the Deposit & Surface Analysis Test Kit (LuminUltra Technologies Ltd., Fredericton, E3G 6M1 NB, Canada) (Supplementary Materials). To facilitate the interpretation, the unit of ATP was converted to microbial equivalent (ME) according to the manufacturer’s instructions. Potential oxygen release during the toxicity tests caused by the NC10 phylum bacteria was checked using a Mettler Toledo dissolved oxygen probe (InPro6860i/12/120/nA, Columbus, 43240 OH, USA) and an M400 transmitter (Supplementary Materials). Total copper in the filtrate (0.22 µm syringe filter) was analyzed with an Inductively Coupled Plasma Mass Spectrometer (ICP-MS NexION 350D, PerkinElmer, Waltham, 02451 MA, USA) following a method reported elsewhere [42].

### 2.6. Statistical Analysis

The Mann–Whitney U test was applied in the comparison of methane consumption, nitrate/nitrite utilization, and ATP among different groups at a significance level of 0.05. The alpha diversity of each sample was calculated according to the equation below for metagenomic data at the genus level.

$$\mathrm{Shannon}\;\mathrm{index}\;(H)=-\sum_{i=1}^{n}p_{i}\times lnp_{i}$$

where *p* is the proportion of individuals of one specific OTU/pathway divided by the total number of individuals and *n* is the number of OTU/pathways.

## 3. Results and Discussion

### 3.1. Toxicity Tests and Copper Speciation

#### 3.1.1. Enrichment and Variations in DAMO Activity

**Figure 1:**
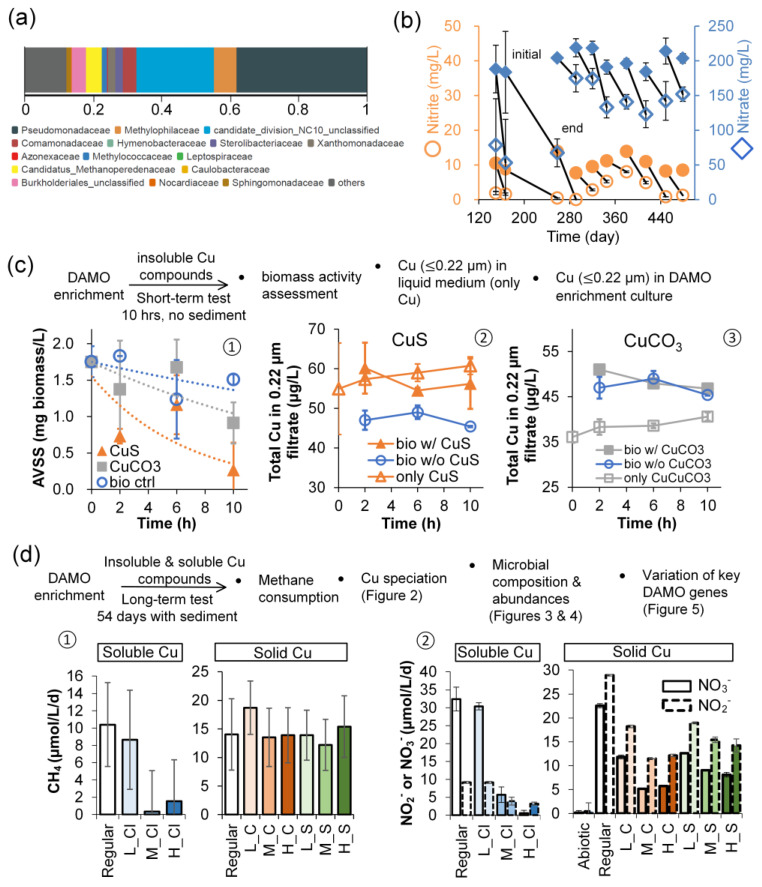
Characterization of the DAMO microorganisms in the enrichment phase and some of the results from the toxicity tests. (**a**) shows the microbial composition at the family level at the end of the long-term enrichment before the test with copper. (**b**) shows the apparent denitrification activity during enrichment. (**c**) shows the results from the 10-hour toxicity test, including the AVSS levels and the variation of the practically soluble Cu fraction. (**d**) shows the results from the 54-day toxicity test, including methane consumption in the presence of CuCl_2_ (L_Cl, M_Cl, and H_Cl, correspond to low, medium, and high levels), malachite (L_C, M_C, and H_C) and covellite (L_S, M_S, and H_S) and the corresponding nitrate/nitrite consumption rates. The colors were applied to distinguish different copper compounds and their levels. Error bars represent one standard deviation (SD) from the mean.

The microbial composition estimated by metagenomic analysis showed that NC10 phylum bacteria account for 22.7% and *Ca*. Methanoperedenaceae account for 4.5% of the total (Figure 1a). At the species level, three commonly reported DAMO bacteria were detectable: *Ca*. Methylomirabilis limnetica, *Ca*. Methylomirabilis lanthanidiphila, and *Ca*. Methylomirabilis oxyfera. Regarding DAMO archaea, *Ca*. Methanoperedens nitroreducens, *Ca*. Methanoperedens sp. BLZ1/BLZ2, and some unknown ANME-2 cluster archaea were identifiable. According to the nitrate and nitrite consumption rates in the last hundred days during the enrichment phase (Figure 1b), the enriched culture showed an estimated apparent methane oxidation rate of approximately 20 ± 9 µmol CH_4_/L/d, which is in the range of commonly reported values in freshwater ecosystems and paddy fields [12].

The short-term tests with the enrichment in the presence of solid copper showed that CuS and CuCO_3_ affected the DAMO activity differently in terms of AVSS (Figure 1c (①)), with CuS being a more potent inhibitor. Copper ions are often thought to be the reason for copper-induced toxicity due to the oxidative stress by in vivo Fenton chemistry or the iron displacement by copper from [4Fe-4S] clusters, leading to the inactivation of essential enzymes in cell metabolism [43]. Here, the copper fraction passing the 0.22 µm filter is chosen to be representative of the soluble fraction in a practical sense (theoretically spans the dissolved/ionic and colloidal size ranges). The comparison of copper ≤ 0.22 µm showed that the biotic CuS group contained 9.8 µg/L more copper than the regularly cultivated group, while the biotic CuCO_3_ system showed negligible difference (Figure 1c (②,③)). Additionally, the results seem to indicate that CuCO_3_ can bind more dissolved Cu^2+^ originally in the medium (Figure 1c (③)). Since dissolution yielded no more than 5 µg/L of Cu^2+^ in both CuS and CuCO_3_ systems over 10 h, it is possible that other copper species of size ≤ 0.22 µm are responsible for the immediate inhibitory effect of CuS. Noticeably, the inhibitory effect on AVSS may result from the potential toxicity of CuS and CuCO_3_ particles, but it is currently difficult to exclude the involvement of dissolved and colloidal copper at the solid–cell interface.

**Figure 2:**
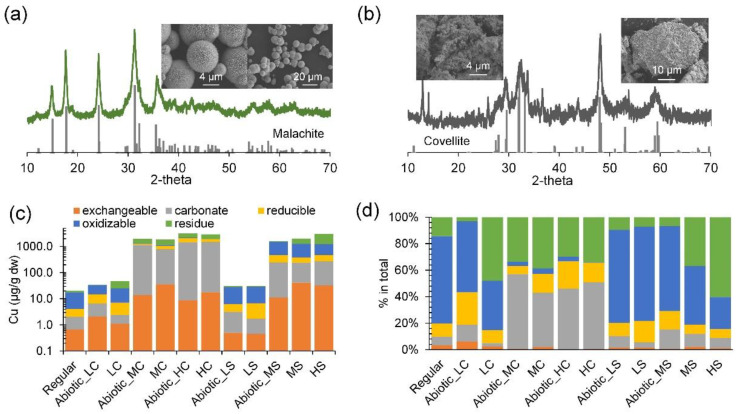
X-ray diffractograms and images from scanning electron microscopy of the synthesized copper compounds and the speciation of copper at the end of the 54-day tests. (**a**) malachite (Cu_2_CO_3_(OH)_2_, simplified as CuCO_3_) and (**b**) covellite (CuS). The standards were added below the diffractograms, and the database codes of covellite and malachite are amcsd 0010981 and amcsd 0009305, respectively. (**c**,**d**) show the sequential extraction results in absolute mass concentrations and proportions, respectively.

Upon ascertaining the inhibitory effect induced by copper (i.e., soluble and colloidal copper released, or even solid itself), to better demonstrate DAMO activity in a copper-contaminated sedimentary environment, copper compounds at the frequently reported levels in natural and contaminated sites (1–80 mg Cu/L) [44,45] were further examined with paired solid and soluble copper in a system containing sediment. The long-term results after 54 days showed that soluble copper from CuCl_2_ significantly hindered methane oxidation and denitrification (Figure 1d (①)). High levels of Cu^2+^, 50 or 80 mg/L, were enough to cease DAMO activity. However, the equivalent dosage of insoluble copper compounds (Figure 2a,b) did not significantly inhibit the apparent methane consumption (Figure 1d (①): test groups are not significantly different from the regular group, *p* > 0.05), except for the low CuCO_3_ group, which was even slightly higher than the normal group (*p* < 0.01) (Figure 1d (①)).

A comparison of apparent nitrate reduction rates showed that the nitrate reduction rate decreased with copper dosage, whether the added copper was in the soluble or solid phase. In the CuS group, the apparent nitrate utilization rates decreased from 22.6 µmol NO_3_^−^/L/d to 9.1 (medium, 59.7% lower than the regular group) and 8.1 (high) µmol NO_3_^−^/L/d; in the CuCO_3_ group, the rate dropped to 5.1 (medium, 77.4% lower than the regular group) and 5.7 (high) µmol NO_3_^−^/L/d (Figure 1d (②)). The overall nitrite reduction showed a similar inhibited trend, but the apparent nitrite utilization seems to be less affected by copper (dropped by 46.6–60.2%), as compared to nitrate reduction. The final nitrite concentrations ranged from 0.3 to 1.1 mg NO_2_^−^/L, suggesting that DAMO bacteria or coexisting nitrite-reducing microorganisms actively consumed nitrite.

If the stoichiometric relationships of nitrate- and nitrite-dependent methane mineralization (Equations (1) and (2)) are considered, and we assume all the reduced nitrate and nitrite are due to methane oxidation, theoretically, maximum methane mineralization can be estimated. It was found that the theoretical estimates, 17–32 µmol, can only account for 45–82% of the measured methane consumption. Since we already considered the loss via possible leakage by subtracting the decline in the abiotic controls from the respective biotic groups, complete mineralization of the consumed methane should not have occurred, and some of the methane may have participated in other microbiological processes. It is also reasonable that such stoichiometry should only hold for the DAMO process over a relatively short period. Potential explanations will be discussed in Section 3.2 and Section 3.3.

(1) $$CH_{4}+4NO_{3}^{-}\to CO_{2}+4NO_{2}^{-}+2H_{2}O\;\Delta G^{o}=-521.4\;\;J/\mathrm{mol}\;{\mathrm{CH}}_{4}$$

(2) $$3CH_{4}+8NO_{2}^{-}+8H^{+}\to 3CO_{2}+4N_{2}+10H_{2}O\;\Delta G^{o\prime }=-928.8\;\mathrm{KJ}/\mathrm{mol}\;{\mathrm{CH}}_{4}\;(pH=7)$$

#### 3.1.2. Variation of Copper Speciation

Copper speciation analysis showed that the primary copper fractions generally comply with the copper phase used (Figure 2 and Figure S1). Specifically, the carbonated and oxidizable fractions were the major fractions in the CuCO_3_ and CuS systems, respectively. The final pH was similar among all the tested groups (7.73–7.79, Figure S2). The commonly thought most toxic fraction, the exchangeable fraction, was 17.7–35.5 µg/g dw and 32.3–41.0 µg/g dw in the CuCO_3_ and CuS systems, respectively, at the end of the tests (Figure 2c,d). Note that the exchangeable fraction here contains free Cu^2+^ as well as Cu^2+^ previously bound to colloidal sediment particles or colloidal CuS/CuCO_3_, but the exact proportion of free Cu^2+^ in the exchangeable fraction was not determined. Additionally, the sequential copper extraction results (Figure 2c,d) also showed that the residual fraction in the medium- and high-level groups, especially CuS, and the low-level CuCO_3_ all increased after the 54-day test, as compared to the corresponding controls. This shift in speciation suggests that dissolution occurred. In the CuCO_3_ systems, dissolution in the presence of microbial activity seems to be less significant than in the CuS groups (Figure 2d).

For denitrifiers in sedimentary environments, contrasting evidence exists for the effect of not only copper but heavy metals in general [46]. For instance, copper of 2–100 µg/g dw was found to have no significant effect or even to enhance denitrification in wetland and freshwater sediments [47,48], while similar levels of copper significantly inhibited denitrification in intertidal sandy sediment [49]. A similar contrasting effect of copper also exists for denitrifiers in soil environments [50,51].

On the contrary, the threshold copper level that may inhibit common denitrifying and DAMO microorganisms seems to lie somewhere between 0.6 and 1 mg Cu^2+^/L in a bioreactor environment. Specifically, previous bioreactor studies on the effect of copper on DAMO showed that Cu^2+^ concentration in the range of approximately 63.5 to 635.5 µg/L supported a quite stable apparent nitrite-DAMO rate (i.e., specific rate, as normalized by 16S rRNA gene copies of the NC10 bacteria) [26]. Copper over 0.6 mg/L had an obvious negative effect on DAMO activity [26,27]; however, sometimes a Cu^2+^ concentration of 1 mg/L had no obvious negative effect [52]. AOM (anaerobic oxidation of methane) coupled with perchlorate reduction was shown to be enhanced when Cu^2+^ concentration increased from 0.06 mg/L to 0.64 mg/L [53]. Moreover, heterotrophic denitrifiers in wastewater treatment facilities have been demonstrated to be sensitive to copper concentration, and 0.95 mg/L of copper was reported to induce a 50% decline in heterotrophic denitrification [54].

The exchangeable fraction in this study, if converted to the equivalent free Cu^2+^ (6 g of sediment slurry with 29% dry content), corresponds to 0.6–1.4 mg Cu^2+^/L. It seems that the enriched DAMO culture is more sensitive to copper than denitrifiers in wastewater treatment facilities and the reported freshwater environment. Besides, the overall methane oxidation in a more realistic system during the 54-day test showed no obvious decline up to an exchangeable copper level of 1.4 mg/L, implying great resilience of the microorganisms in a complex environment.

### 3.2. Shift in Microbial Composition

#### 3.2.1. Overall Microbial Composition Changes

**Figure 3:**
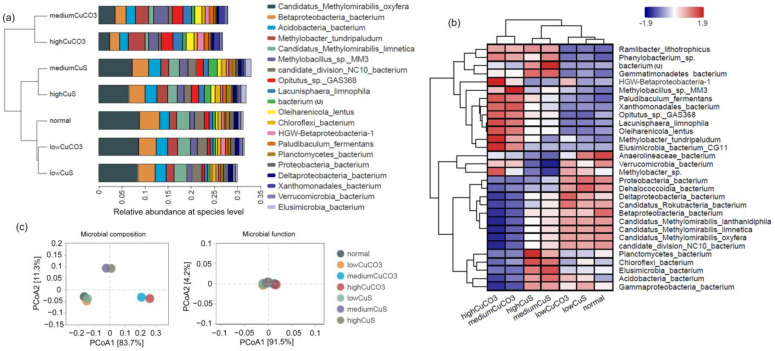
Cluster analysis of the control and test groups. (**a**) shows hierarchical clustering analysis by UPGMA on the left and relative abundance at the species level on the side (top 20 most abundant). (**b**) shows the cluster analysis and heatmap of the microbial community composition of each sample with the 30 most abundant species. The species that could not be assigned was labeled with “u” in parentheses. (**c**) PCoA calculated with Bray–Curtis distance on the microbial composition and functions based on relative abundance from KEGG Orthologue (Enzyme) profiling. Distances between symbols on the ordination plot reflect relative dissimilarities in community structures/functions. The variation explained by each PCoA axis is given in parentheses.

The phylogenetic composition of the samples after the 54-day test indicates the *Proteobacteria* and NC10 phyla constitute the vast majority of the enriched DAMO microbiota in all but the medium- and high-level CuCO_3_ groups (Figure S3). The proportion of microorganisms from the *Proteobacteria* and *Verrucomicrobia* phyla exceeded the NC10 phylum upon the treatment of medium- and high-level CuCO_3_. In DAMO systems, *Verrucomicrobia* have often been detected and may have performed partial denitrification or dissimilatory nitrate reduction to ammonium [55], but their exact role is to be investigated. At the species level, *Ca*. Methylomirabilis oxyfera and *Ca*. Methylomirabilis limnetica from the NC10 phylum were still dominant among the identifiable group, while *Ca*. Methylomirabilis lanthanidiphila may have merged with other relevant microorganisms and appeared as candidate division NC10 bacterium, as shown in Figure 3a. Specifically, among all the analyzed groups in the 54-day tests, the regularly cultivated and low-level solid copper groups had the highest relative abundance of NC10 phylum bacteria, ranging from 13.2% to 13.8%. The medium (11.3%) and high CuS (10.2%) groups had less abundant NC10 division bacteria while the abundance in the medium (5.5%) and high CuCO_3_ (3.7%) groups was the lowest. It is noticeable that the relative abundance of these representative DAMO archaea, such as *Ca*. Methanoperedens nitroreducens, significantly declined and disappeared from the list of major taxa.

PCoA (Principal Coordinates Analysis) based on species-level composition and functions (Figure 3c) showed that low levels of solid copper did not induce a significant change of the microbial composition, while high and medium levels of solid copper led to distinct and separated clusters, which corroborates the observation from Figure 3b. Function-wise, the tested groups showed relatively high similarity (Figure 3c). Alpha diversity analysis based on metagenomic reads at the genus level revealed a slightly higher Shannon diversity in the copper-dosed groups, and the index generally increased with the level of solid copper (normal = 3.26; L, M, H CuS = 3.39, 3.60, 3.69; L, M, H CuCO_3_ = 3.33, 3.94, 4.04).

Some of the enriched species in the medium and high CuCO_3_ groups after the 54-day incubation include *Methylobacter tundripaludum*, *Methylobacillus* sp. MM3, *Opitutus* sp. GAS368, and *Paludibaculum fermentans*. In contrast, the medium- and high-level CuS seem to have enriched the *Planctomycetes* and *Chloroflexi* bacteria (Figure 3b).

*Methylobacillus* sp. MM3 is a methanol-utilizing methylotroph that carries genes encoding various methanol dehydrogenases and all genes required in denitrification [56]. Another detected species, *P. fermentans*, is known to grow in anoxic conditions and couple the fermentation of sugars with the reduction of nitrate to nitrite. *P. fermentans* cannot utilize methanol or acetate and cannot reduce nitrite [57]. *Planctomycetes* and *Chloroflexi* spp. microorganisms have been commonly detected in nitrogen-removing facilities and were found to live off the compounds released during cell lysis, primarily through fermentation. Species belonging to the phylum *Chloroflexi* have been shown to conduct nitrate/nitrite respiration [58]. Analysis of the relevant nitrate-reduction (*narGHI*) and nitrite-reduction (*nrfAH*) genes from the *Chloroflexi* bacterium in dissimilatory nitrate reduction to ammonium all showed increased abundance with the dosage of CuS (Figure S4), implying these microorganisms might have coupled the oxidation of available reductive substances and denitrification. Another enriched group, *Elusimicrobia* bacteria, are fermenters and can also conduct denitrification using nitrate/nitrite [59].

**Figure 4:**
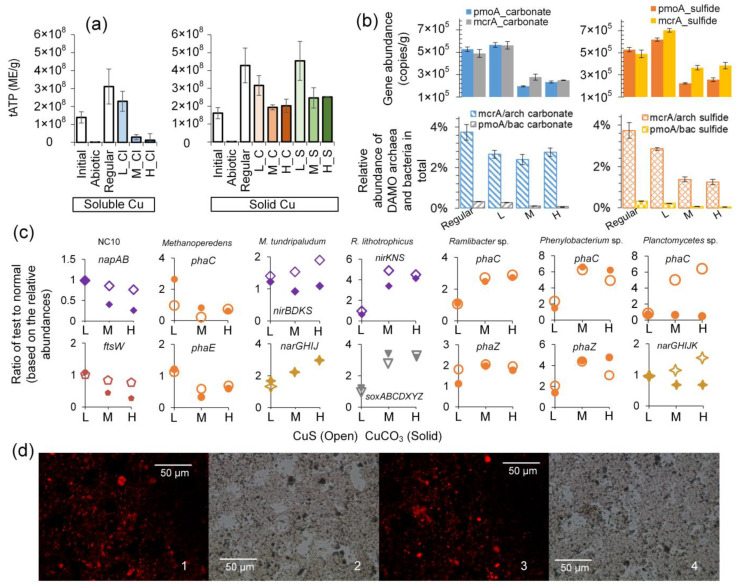
Total ATP concentrations, the qPCR results, the variation of representative genes from some dominant microorganisms in the 54-day test, and the Nile Red staining results. (**a**) shows tATP before and after the 54-day test; the colors were applied to distinguish different copper compounds and their levels. (**b**) shows the abundance of genes targeting DAMO microorganisms and general bacteria and archaea after the 54-day test. (**c**) shows the variation of representative genes from major taxa as compared to the normally cultivated group. (**d**) shows the representative paired fluorescence and non-fluorescence phase contrast microscopy images of slurries: 1 and 2 from the CuS system and 3 and 4 from the CuCO_3_ system (all with high-level copper). Error bars represent one SD from the mean.

Interestingly, methanotrophs, such as *Methylobacter tundripaludum*, were found to be more abundant in the medium and high CuCO_3_ groups. *M. tundripaludum* may be able to couple methane oxidation to denitrification because the transcripts of *narG*, the gene that encodes nitrate reductase, were detected in a metatranscriptome study [60], and *M. tundripaludum* also carries the *nirS* gene required in dissimilatory nitrite reduction to NO [61]. These lines of information suggest that alternative denitrification other than that conducted by representative DAMO microorganisms may have occurred. The *narGHIJ* genes for nitrate reduction and *nirBDKS* genes for nitrite reduction from *M. tundripaludum* generally showed increased abundance with solid copper (Figure 4c), suggesting that this species can better tolerate copper toxicity and probably had partly taken over the methanotrophic activity previously conducted by those representative DAMO microorganisms.

Furthermore, *M. tundripaludum* is capable of aerobic respiration. It is also known that *Ca*. Methylomirabilis oxyfera can release a tiny amount of oxygen when the activity of particulate methane mono-oxygenase is low [10], and oxygen release via a similar mechanism has also been speculated for *Pseudomonas aeruginosa* (ca. 10 ppb oxygen released) [62]. Although exogenous oxygen intrusion is unlikely after the repeated vacuum and refilling cycles and with incubation under a methane/nitrogen atmosphere at positive pressure, the inhibition caused by solid copper may result in oxygen release from the NC10 bacteria. This internally released oxygen in the medium- and high-level solid copper systems may have encouraged the aerobic methane oxidation by *M. tundripaludum*. Therefore, we checked possible oxygen generation inside the bottles; however, the dissolved oxygen was lower than the detectable limit, approximately 0.6 ppb according to the probe used, upon medium-level copper addition, which is shown here to inhibit DAMO enrichment. Nevertheless, it must be acknowledged here that the release of oxygen might be gradual and span a much longer time than the monitoring period (maximum 48 h, as shown in Supplementary Materials) and thus complicate the dissolved oxygen measurement. If there is any oxygen released from *Ca*. Methylomirabilis oxyfera and other representative DAMO bacteria, that oxygen could help explain the discrepancy in theoretical and measured methane consumption. Overall, our results generally support the involvement of *M. tundripaludum* in methane consumption or even in DAMO.

#### 3.2.2. Variation in Representative DAMO Microorganisms

Although relative abundances, as shown in Figure 3a,b, are frequently applied to reveal the variation of dominant taxa, the data often cannot reflect the actual variation of target microorganisms [63]. Here, the variation of DAMO microorganisms is further examined based on two other sources, namely the total ATP (tATP), a marker of viable cells (i.e., mainly from living cells), and the qPCR assay. It is noteworthy that uncertainty is inherent in these assays because dead/dying cells also contribute, though marginally, to tATP, and it is challenging for qPCR to differentiate genes from live and dead cells [64,65].

Quantification of tATP suggests that the total number of microorganisms, as expressed by ME (microbial equivalent), has doubled in the normal (1.6 × 10^8^ to 4.3 × 10^8^ ME/g) and low-level solid copper groups (Figure 4a), implying that the carbon and energy from methane was assimilated into biomass. This should explain the discrepancy in theoretical estimation according to the complete mineralization discussed in Section 3.1.2. In terms of active biomass, low-level CuS (4.5 × 10^8^ ME/g) seems to have a similar effect to low-level CuCO_3_ (4.3 × 10^8^ ME/g). Compared with medium- and high-levels of CuCl_2_, which lost over 90% of the microorganisms (Figure 4a), the levels of active microorganisms in medium and high CuCO_3_ groups are similar to the initial level, while those in the medium and high CuS groups are even a little higher than the initial level, ranging from 1.9 × 10^8^ to 2.5 × 10^8^ ME/g (Figure 4a).

Regarding total bacteria and archaea, conversely, qPCR results showed an overall increasing trend in the presence of solid copper (Figure 4b and Figure S5). The total microorganisms based on 16S rRNA genes in the CuS systems are 1.3–1.5 times those in the CuCO_3_ systems, and all are in the range of 2.2 to 5.0 × 10^8^ copies/g. Since tATP quantification was checked and confirmed to not be affected by copper compounds, this contrast implies that the regular qPCR assays here must have captured a significant number of genes from dead cells or the extracellular space. As for the two representative DAMO microorganisms, the abundances of *pmoA* and *mcrA* genes are more consistent with the tATP quantification, showing that the low copper load induced minimal or even no inhibitory effect, while the medium and high copper loads greatly inhibited the growth of the two representative DAMO microorganisms (Figure 4b). *Candidatus* Methanoperedens spp. seem to be less affected by CuS than CuCO_3_, and *Candidatus* Methylomirabilis spp. responded to the two compounds similarly. Low levels of solid copper generally encouraged the growth of these microorganisms, especially in the CuS groups (Figure 4b). Specifically, *mcrA* and *pmoA* gene abundances were 4.9 × 10^5^ and 5.3 × 10^5^ copies/g, respectively, in the regular group and increased to 7.0 × 10^5^ and 6.2 × 10^5^ copies/g, respectively, in the low CuS group. The *mcrA* and *pmoA* gene abundances dropped to 2.0 × 10^5^ to 2.8 × 10^5^ copies/g in the medium- and high-level copper groups, except the *mcrA* in medium and high CuS groups, which were 3.6 × 10^5^ and 3.8 × 10^5^ copies/g, respectively (Figure 4b).

### 3.3. Effects of Copper on Major DAMO Pathways

**Figure 5:**
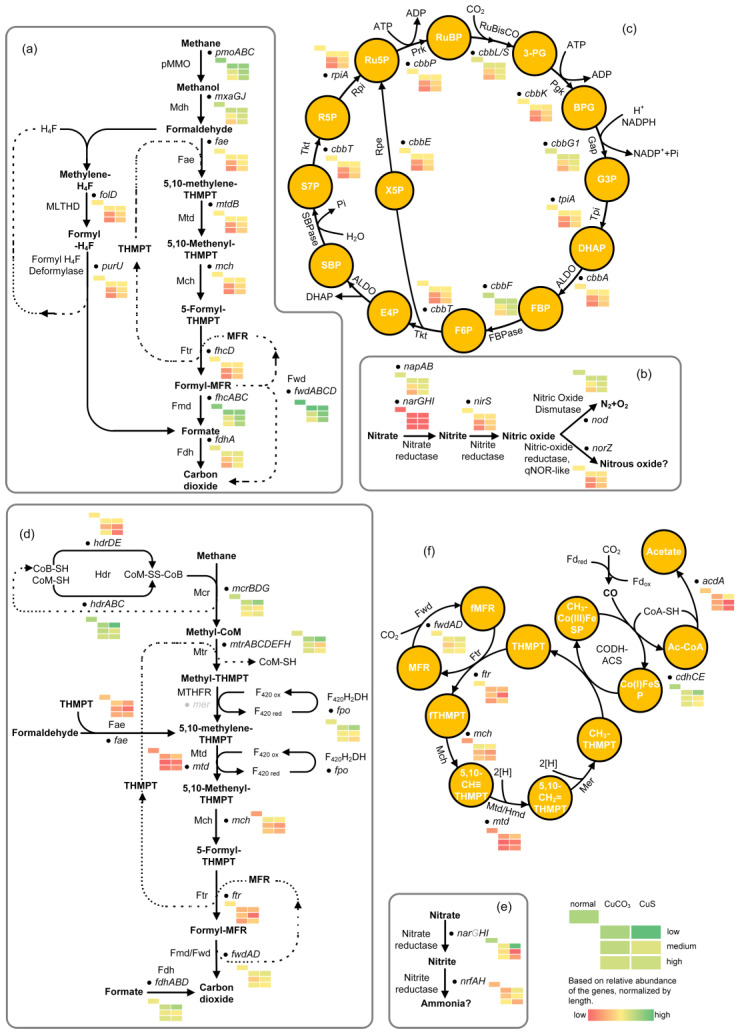
The metabolic pathway and relative abundance of key genes of DAMO bacteria (**a**–**c**) and DAMO archaea (**d**–**f**), with predicted metabolic function as recorded in KEGG. Gray color indicates the absence of the enzyme or complex. The abbreviations and the corresponding full names are as follows: (**a**) Mdh: methanol dehydrogenase, Fae: formaldehyde activating enzyme, Mtd: methylene-tetrahydromethanopterin dehydrogenase, Mch: methenyl-tetrahydromethanopterin cyclohydrolase, Ftr: formyltransferase, Fmd: formyl-methanofuran dehydrogenase, Fdh: formate dehydrogenase, H4F: tetrahydrofolate; THMPT: tetrahydromethanopterin, MFR: methanofuran; (**b**) enzyme names spelt out; (**c**) 3-PG: 3-phosphoglycerate, Pgk: phosphoglycerate kinase, BPG: 1, 3-byphosphate glyceric acid, Gap: glyceraldehyde 3-phosphate dehydrogenase, G3P: glyceraldehyde 3-phosphate, Tpi: triosephosphate isomerase, DHAP: dihydroxyacetone phosphate, ALDO: fructose-bisphosphate aldolase, FBP: fructose 1,6-bisphosphate, FBPase: fructose 1,6-bisphosphatase, F6P: fructose 6-phosphate, Tkt: transketolase, E4P: erythrose 4-phosphate, SBP: sedoheptulose 1,7-bisphosphate, SBPase: sedoheptulose bisphosphatase, S7P: sedoheptulose 7-phosphate, R5P: ribose 5-phosphate, Rpi: ribose 5-phosphate isomerase, Ru5P: ribulose 5-phosphate, Prk: phosphoribulokinase, RuBP: ribulose 1,5-bisphosphate; (**d**) Mcr: methyl coenzyme M reductase (the *mcrA* was not identified), Mtr: methyltransferase, MTHFR: methylenetetrahydrofolate reductase, Fmd/Fwd: formyl-methanofuran dehydrogenase, Hdr: heterodisulfide reductase; (**e**) enzyme names spelt out; *narG* was not identified; (**f**) Co(I)FeSP: Co(I) corrinoid Fe-S protein, Co(III)FeSP: methyl-Co(III) corrinoid Fe-S protein, fMFR, formylmethanofuran, CODH-ACS: carbon monoxide dehydrogenase/acetyl-CoA synthase.

The relative abundances of central genes involved in methane oxidation, denitrification, and the carbon assimilation of DAMO microorganisms were analyzed to reveal more function-related changes in different copper conditions. Generally, the pattern of relative abundance of these central genes agrees with the variation in tATP and the relative abundance of representative DAMO microorganisms revealed by qPCR (Figure 4a,b). Low levels of copper led to the comparable or even higher relative abundance of key genes in DAMO pathways, as compared to the control group (Figure 5). The relative abundances of these key genes declined with increasing copper load.

Specifically, the relative abundance of genes pertinent to bacterial methane oxidation, such as the *pmoABC*, encoding the pMMO, were less affected in the CuS groups (Figure 5a). Besides, the relative abundance of genes encoding key enzymes including ribulose-1,5-bisphosphate carboxylase/oxygenase (RuBisCo) in the Calvin–Benson–Bassham (CBB) cycle of DAMO bacteria (Figure 5c) were less affected by CuS as well. So, too, was the relative abundance of genes encoding proteins related to cell division of NC10 phylum bacteria (*ftsW*) (Figure 4c) and relevant genes for the denitrification pathway (e.g., *NapAB*, *NirS*) (Figure 5b), suggesting that a relatively lower number of DAMO bacteria in CuCO_3_ systems may be related to the inhibited carbon assimilation and denitrification.

As for the relatively higher *pmoA* gene abundance in the CuS groups, a potential reason could be the less affected alternative methane oxidation pathways in the presence of sulfide as compared to carbonate. We found that the relative abundance of sulfur-metabolizing genes of *Ca*. M. oxyfera, including *moaD*, *thiS*, *soxY*, and *cysK*, were all higher in the groups dosed with medium and high CuS as compared with the corresponding CuCO_3_ groups (Figure S4). These genes are related to folate/thiamine synthesis (KEGG pathway mox04122). Folate is required by prokaryotes as a cofactor for the biosynthesis of a diverse range of cellular components, including tetrahydrofolate. Tetrahydrofolate can serve as a one-carbon carrier in a variety of biosynthetic and degradative processes. DAMO bacteria may have also transformed methane into formaldehyde and followed the tetrahydrofolate pathway (Figure 5a), although future studies are required to check this speculation.

Regarding DAMO archaea, many of the key genes encoding enzymes for methane activation were less abundant in the CuS groups, such as the *hdr* genes for two-electron reduction of coenzyme B-S-S-coenzyme M (CoB-S-S-CoM) and *fpo* for F_420_H_2_ dehydrogenase (Figure 5d). When the key genes involved in the reductive acetyl-CoA pathway, the major carbon assimilation pathway by DAMO archaea, were examined, a similar trend could also be seen, such as the lower abundance of the CODH-ACS gene complex in the CuS groups (Figure 5f). Furthermore, the conversion of acetyl-CoA to acetate seems to be greatly inhibited by CuS (Figure 5f). A similar pattern of inhibition by CuS can be seen in the relative abundance of genes related to archaeal cell division (*ftsZ*) (Figure S4).

Furthermore, DAMO archaea have been reported to synthesize intracellular storage compounds, potentially PHA/PHB (polyhydroxyalkanoate/poly [3-hydroxybutyrate]) in times of nutrient imbalance [66]. Here, the relative abundance PHA/PHB synthesis/degradation-related genes (*phaC* and *phaE*) from DAMO archaea all diminished with increasing copper load (Figure 4c). However, the relative abundance of *phaC* and *phaZ* from other microorganisms, including *Planctomycetes*, *Phenylobacterium*, and *Ramlibacter* spp., all increased 2–6 times as compared to the control without excessive copper (Figure 4c). The Nile Red staining analysis confirmed the presence of PHA/PHB-like compounds in the system (Figure 4d), but we currently have not identified the species that synthesized these compounds. Here, the synthesis of PHA/PHB by *Planctomycetes* and *Phenylobacterium* spp. or DAMO archaea might be out of a protective function against stress induced by copper ions, as discussed elsewhere [67]. Additionally, the intracellular reduced carbon could be the second reason for the discrepancy in theoretical (complete mineralization) and measured methane consumption.

### 3.4. Difference in Energy Content Could Be a Determining Factor

The short-term test showed that CuS was a more potent inhibitor than CuCO_3_, potentially due to the higher level of copper, ≤ 0.22 µm, from CuS. The copper speciation results after the 54-day tests indicate a higher-level dissolution of CuS than CuCO_3_. The variation in apparent denitrification rate at the end of the long-term tests also agrees with the inferred dissolution (Figure 1d (②)). Nevertheless, the quantification of tATP and *pmoA*/*mcrA* gene abundance suggests that CuS affected the DAMO enrichment but to a lesser extent than CuCO_3_, although both compounds at medium and high loads seriously inhibited the microbial metabolism. These lines of evidence suggest microorganisms were able to partly counterbalance the negative effect of Cu^2+^ from CuS dissolution by manipulating the energy gains. In other words, CuCO_3_ may have only served as a source of inhibitor and a less favorable carbon source, while CuS was a less favorable but reachable energy source. The Gibbs free energy gains from CuS oxidation are slightly lower than the corresponding methane oxidation (Equations (3) and (4)). Although the released Cu^2+^ is still an inhibitor, the slow but steady co-released sulfide may have supported the sulfide-based denitrification (Equations (5) and (6)), considering the higher Gibbs free energy gains of S^2−^ oxidation as compared to CuS oxidation. Liquid phase analysis of the tested systems showed that the control without copper addition and the CuCO_3_ groups contained 0.52–0.59 mg SO_4_^2−^/g dw while the high-level CuS group contained 0.79–0.81 mg SO_4_^2−^/g dw, corroborating the oxidation of sulfide from CuS.

(3) $$CuS+4NO_{3}^{-}\to Cu^{2+}+SO_{4}^{2-}+4NO_{2}^{-}\;\Delta G^{o}=-344.1\;\mathrm{KJ}/\mathrm{mol}\;\mathrm{CuS}$$

(4) $$3CuS+8NO_{2}^{-}+8H^{+}\to 3Cu^{2+}+3SO_{4}^{2-}+4N_{2}+4H_{2}O\;\Delta G^{o\prime }=-741.4\;\mathrm{KJ}/\mathrm{mol}\;\mathrm{CuS}\;(\mathrm{pH}=7)$$

(5) $$S^{2-}+4NO_{3}^{-}\to SO_{4}^{2-}+4NO_{2}^{-}\;\Delta G^{o}=-533.8\;\mathrm{KJ}/\mathrm{mol}\;S$$

(6) $$3S^{2-}+8NO_{2}^{-}+8H^{+}\to 3SO_{4}^{2-}+4N_{2}+4H_{2}O\;\Delta G^{o\prime }=-941.2\;\mathrm{KJ}/\mathrm{mol}\;S\;(\mathrm{pH}=7)$$

A relatively high abundance of species from the family *Comamonadaceae*, known to be able to enhance sulfur cycling in various environments [68], supports this speculation. For example, when the relative abundance of genes related to sulfide utilization and denitrification was examined, the gene clusters for nitrate reduction (*napABCDE*, *narHI*) and nitrite reduction (*nirKNS*), as well as sulfur oxidation (*soxABCDXYZ*, *dsrABCFH*) from *Ramlibacter lithotrophicus*, were all more enriched in the high CuS condition (Figure 4c and Figure S4), indicating that these species may have coupled sulfide oxidation to denitrification. Additionally, the relative abundance of sulfur utilization genes by some other species, such as *Methylobacillus* sp. MM3 (*soxADGXYZ*, *fccABC*) and *Betaproteobacteria* bacterium (*dsrA*, *fccAB*) (Figure S4), all increased in the presence of CuS, implying potential sulfide oxidation.

## 4. Conclusions

Malachite (Cu_2_[OH]_2_CO_3_) and covellite (CuS), along with soluble copper, can largely represent three of the common phases of inorganic copper in copper-contaminated freshwater systems. According to the DAMO rates, copper speciation, microbiological parameters, and metagenomic analysis presented here, some major conclusions are as follows: (a) other than the moderate inhibition of denitrification in the presence of low-level Cu_2_[OH]_2_CO_3_ and CuS, copper up to 1 mg Cu/L, soluble or insoluble, showed minimal effect on methane consumption in 54 days; (b) the short-term inhibitory effect from CuS was immediate, likely not from dissolved Cu^2+^ but other copper species with size ≤ 0.22 µm, while that effect from Cu_2_[OH]_2_CO_3_ was milder upon contact with the DAMO enrichment; (c) the medium- and high-level Cu_2_[OH]_2_CO_3_ and CuS induced a pronounced decline in the abundance of representative DAMO microorganisms after 54 days; however, the oxidation of sulfide from CuS can alleviate the toxicity of Cu^2+^ to the DAMO microorganisms, especially *Candidatus* Methanoperedens spp.; (d) some enriched microorganisms upon solid copper treatment, such as *Methylobacter tundripaludum*, might have participated in methane oxidation or even DAMO; (e) the methane consumed in the presence of solid copper may be diverted more to internal storage as opposed to denitrification, rendering incomplete methane oxidation.

Overall, it is reasonable to imply that the growth of representative DAMO microorganisms in a more complex and realistic system previously contaminated by heavy metals may undergo sustained depression. The straightforward positive relationship between nitrogen input to freshwater ecosystems and the abundance of DAMO microorganisms, a frequent observation in previous publications, does not hold in a copper-contaminated sedimentary environment or a sedimentary environment located downstream of a copper-contaminated site, considering the remobilization of contaminant. While methane consumption in a short period may not be hampered by heavy metal precipitates, long-term methane abatement capability may be affected due to the shift of DAMO microorganisms. Based upon this study, field analysis with DAMO microorganisms at their natural abundance levels is necessary to further examine their responses to heavy metal contamination. Furthermore, whether the representative DAMO microorganisms can recover from the adverse circumstance of excessive copper exposure, or heavy metals in general, can be the subject of future studies.

## Data Availability

The raw data supporting the conclusions of this article will be made available by the authors on request.

## Supplementary Materials

The following supporting information can be downloaded at: https://www.mdpi.com/article/10.3390/microorganisms12112259/s1. More details can be found on the following: 1. Enrichment of DAMO microorganisms and relevant computation, 2. IC and GC analyses, 3. Copper compound characterization and speciation analysis, 4. More details on the two toxicity tests, 5. Quantification of potential oxygen release from NC10 phylum bacteria, 6. Quantification of ATP in suspension and slurry samples, and 7. Metagenome sequencing and bioinformatics analyses. Figure S1: Cu levels and speciation after the test with CuCl_2_ on DAMO enrichment; Figure S2: The pH and ORP measured after the 54-day toxicity tests with copper; Figure S3: Cluster analysis of the control and test groups with hierarchical clustering analysis by UPGMA on the left and relative abundance at the phylum and genus level on the side; Figure S4: Variation of representative genes as compared to the normally cultivated group from major taxa; Figure S5: Absolute gene copies of general bacteria and archaea in the tested groups after 54 days. Table S1: qPCR primers and PCR protocols used in this study. Refs. [69,70,71,72,73,74,75,76] can be found in Supplementary Materials.
