# Supplementary material for: The Effects of Model Insoluble Copper Compounds in a Sedimentary Environment on Denitrifying Anaerobic Methane Oxidation (DAMO) Enrichment
Source: Microorganisms. 2024 Nov 7;12(11):2259. doi: 10.3390/microorganisms12112259 (PMC11596795; doi:10.3390/microorganisms12112259)
Supplement: Supplementary file 1 [file microorganisms-12-02259-s001.zip › microorganisms-3238133-supplementary.pdf]

Supplementary materials

The effects of model insoluble copper compounds in a sedimentary environment on denitrifying anaerobic methane oxidation (DAMO) enrichment

Longfei Xia [1,2,†], Yong Wang [1,3,†], Peiru Yao [1,3], Hodon Ryu [4], Zhengzhong Dong [1,3], Chen Tan [1,3], Shihai Deng [1,3], Hongjian Liao [1] and Yaohuan Gao [1,3,*]

[1] School of Human Settlements and Civil Engineering, Xi'an Jiaotong University, 19-3027 iHarbour Campus, Xi'an 710115, China;
[2] Shaanxi Provincial Land Engineering Construction Group, Xi'an 710075, China
[3] Institute of Global Environmental Change, Xi'an Jiaotong University, 19-3027 iHarbour Campus, Xi'an 710115, China
[4] United States Environmental Protection Agency, Office of Research and Development, Cincinnati, 45268 OH, USA; ryu.hodon@epa.gov
* Correspondence: gaoyaohuan@xjtu.edu.cn
† These authors contributed equally to this work.

Description of the material:

- 12pages
- 5 figures
- 1 table
- Text on details of
  1. Enrichment of DAMO microorganisms and relevant computation
  2. IC and GC analyses
  3. Copper compound characterization and the speciation analysis
  4. More details on the two toxicity tests
  5. Quantification of potential oxygen release from NC10 phylum bacteria
  6. Quantification of ATP in suspension and slurry samples
  7. Metagenome sequencing, and bioinformatics analyses

1. Enrichment of DAMO microorganisms and relevant computation

During the enrichment, the bottles were sealed with rubber stoppers and aluminum crimp caps, vacuumed and filled with ultrapure N_2 at least three times, and then vacuumed and filled with ultrapure methane another three times and the final headspace pressure was set at 0.14 MPa. Incubation was carried out with bottles in their inverted position in a dark condition (aluminum foil wrap) on a shaking table (Shiping sph300, China) at 50 rpm.

The pressure in the headspace of bottles was measured with a precision pressure transducer (MIK-P3000, ±0.075% accuracy). Partial pressure of methane was calculated based on the headspace composition as quantified by the gas chromatography while the dissolved methane was estimated based on Henry's Law (K_H=0.0013 mol/L·atm). Besides, assuming methane in the headspace behaves like an ideal gas, we can obtain the moles of methane (n=PV/RT) in the headspace based on the headspace volume (165 ml during enrichment and 66.5 ml in the other tests). The total methane in an individual bottle is the sum of headspace methane and dissolved methane.

2. IC and GC analyses

Approximately 40 ml of the liquid medium was exchanged every 15-20 days to replenish the essential minerals and also accelerate the acclimation of DAMO microorganisms. The level of residual nitrate/nitrite was quantified with an ion chromatograph (Dionex Integrion, USA) [1]. For ion analysis, the liquid samples passed 0.45 μm syringe filters and then the IC-RP10 Cartridge (Bonna-Agela Technologies, USA), respectively. Both sampling and medium exchange were conducted in a glove box. After medium exchange, the bottles underwent the same gas exchange as mentioned before. Note that our system is sealed by thick rubber stoppers and aluminum caps to avoid gas exchange so that we do not continuously supply methane to the bottles.

Methane gas analysis was set up with both the TCD and FID systems as a direct injection method. The analysis during the enrichment phase was mostly done with TCD while the analysis during the toxicity tests was mostly done with the FID. As for the GC-TCD method, an HP Plot Q column (30 m×0.530 mm×40 μm) was used. Helium was the carrier gas and the injection volume was 50 μl. The temperature of the injection port and the detector was 60 °C and 250 °C, respectively. The temperature program was 50 °C for 5 min, then increased with a rate of 50 °C/min till 140 °C and held there for 2 min. The flow rate inside the column was 2 ml/min. The GC-FID method applied an HP-5 column (30m×320μm×0.25μm). Helium was also the carrier gas. The temperature of the injection port and the detector was 50 °C and 250 °C, respectively. Oxygen and hydrogen gases were generated on-site with a TP-3220 oxygen generator and a TP-3030C hydrogen generator (TP Instrument, Beijing), respectively. The temperature in the oven was 40 °C for 0.5 min and then increased at 3 °C/min till 55 °C and then increased at 80 °C/min till 290 °C.

3. Copper compound characterization and the speciation analysis

The solid copper phases, basic copper carbonate ($Cu_2[OH]_2CO_3$, simplified hereafter as $CuCO_3$) and copper sulfide (CuS), were synthesized following the stoichiometric relation for the respective precipitation reactions with $CuCl_2$ and Na_2CO_3 or Na_2S. The copper compounds synthesized were characterized with XRD and SEM according to methods reported elsewhere [2], except that a Bruker D8 Advance X-ray Diffractometer was used. SEM was carried out with a ZEISS Gemini SEM 500 under an accelerating voltage of 15 kV.

The Tessier sequential extraction sequential extraction method was adapted from elsewhere [3-5]. Specifically, ① the exchangeable fraction (including water soluble), ② the carbonate fraction, and ③ those associated with Fe/Mn oxides (reducible as shown in the text) were extracted using 1 mol/L of $MgCl_2$, 0.11 mol/L of acetic acid, and 0.5 mol/L of ammonium hydroxide (pH=1.5), respectively [3]. The solid after the last extraction was digested with 30% H_2O_2, 0.02 mol/L HNO_3, and then treated with 1 mol/L ammonium acetate (pH=2) for ④ the fraction binds to organics and sulfide (oxidizable as shown in the text) [3]. The residual solids were acid-digested with aqua regia and HF for ⑤ the residual fraction. All extraction solutions were filtered (0.22 μm) for Inductively Coupled Plasma Mass Spectrometer analysis with a NexION 350D ICP-MS (PerkinElmer, USA) following a method published elsewhere [2].

4. More details on the two toxicity tests

(1) the 10-hour test

This test was conducted in 100 ml glass bottles (the exact volume is around 120 ml). Each bottle contained 50 ml medium (the same composition as mentioned in the main text) and received 1 ml of inoculum from a mother reactor used for enrichment. Two stock medium solutions, one with 50× concentrated copper solid (sonicated to disperse the particles) and the other regular, were purged by ultrapure nitrogen gas beforehand and the solution as well as the 100 ml glass bottles were then transferred into a glove box. The solutions underwent further gas exchange during the transfer process. Once inside the glove box, a certain volume of the regular medium was added to each bottle and then 1 ml of inoculum was added. The medium that contained the copper compound was thoroughly mixed and then added to each bottle at last to make a final copper level of 1 mg Cu/L. The test bottles were then sealed with rubber stoppers and aluminum crimp caps. The bottles were quickly transferred outside and the headspace was exchanged by nitrogen gas with a vacuum system two times and then ultrapure methane. The negative control contained a regular medium with inoculum while the test bottles were supplemented with either CuS or $CuCO_3$ powders. Each condition had two replicates and the bottles were left on a shaking table (50 rpm) upside down at

room temperature with aluminum foil wrap to protect them from light.

After zero (after headspace gas exchange), 2 hours (since the contact of copper and the microbial cells), 6 hours, and 10 hours, the solution inside the corresponding bottles was mixed by handshaking and sampled for ATP analysis (QG21W-50C for wastewater and suspended growth system, LuminUltra Technologies Ltd., Canada) and filtration (0.22 µm) for "dissolved" fraction of copper in the liquid phase. The opened bottles were sacrificed after sampling. Note that any copper passing through the 0.22 µm filters is considered "dissolved" in our study and the term "dissolved" does not mean the copper fraction is all in an ionic state. Tiny CuS and $CuCO_3$ particles and the colloidal fractions as well as the true ionic copper could all exist in the filtrate. Here, the focus is on the comparison of copper content in the controls (started with regular medium) and test groups because the contrast can give some clues to the toxicity of CuS and $CuCO_3$ solid particles at the initial stage of dissolution. Exactly which form of copper is responsible for the revealed toxicity can hardly be known and the topic is out of the scope of the current study.

(2) the 54-day test

The 54-day tests were conducted in 100 ml bottles with a solid and liquid volume of ca. 55 ml (6 g slurry with 50 ml liquid, average solid content of the slurry was 29%) and a 66.5 ml headspace. Each copper condition has three replicates. All the liquids were purged by ultrapure nitrogen gas for at least 30 min before they were moved into a glove box. Solid copper suspensions were sonicated and purged with nitrogen gas before they were moved inside the glove box. Mixing was carried out inside the glove box and the bottles were sealed with thick rubber stoppers and aluminum crimp caps.

After the bottles were transferred out from the glove box, each bottle underwent three cycles of vacuuming and nitrogen filling. Controls were prepared as the test groups except that they were autoclaved for 30 min at 121 ℃. The bottles were then inverted and left on a shaking table, covered with aluminum foils. The headspace methane was sampled on the second day for a pseudo-equilibrium gas-phase methane level, as the initial value, and then every three days onwards. Total methane was computed based on Henry's Law [6]. The gas sampling volume was 50 µL (Trajan SGE 005250, Australia).

The pH of the liquid phase was measured with a Mettler Toledo pH meter (FE28) while the slurry ORP was quantified with a Hach MTC10103 probe connected to a Hach HQ40d Meter. The NO_3^-/NO_2^- and SO_4^{2-} were measured with IC as previously mentioned. Copper speciation was carried out as mentioned in the main text.

The denitrification rate was calculated based on the quantification using IC. Nitrate reduction rate was based on the decrease of nitrate level while nitrite reduction rate was based on the net decrease of nitrite (difference between initial and final) and the decrease in nitrate (nitrate to nitrite was the first step).

5. Quantification of potential oxygen release from NC10 phylum bacteria

Measurement was conducted in a 100 ml gas-tight glass electrolysis cell (Gaoss Union) with a 90 ml enriched DAMO culture of the same origin as the other tests. The liquid inside the cell was thoroughly purged with nitrogen gas until the meter showed no detectable DO concentration. In one case, after nitrogen purging, the sealed cell and probe assembly were put into an anaerobic box and measurement was confirmed in an absolute oxygen-free atmosphere. Soluble copper (30 and 60 mg Cu/L) was dosed at the beginning of the incubation. Microbial cells were collected from a continuously running bioreactor and each time 120 ml of suspension was used to concentrate (8000 rpm, 5 min) and inoculate the electrolysis cell. The validity of the probing system was confirmed by continuously but slowly purging an air-saturated base medium with UHP nitrogen and then reversing the procedure for oxygen detection. The detection limit was approximately 0.6 ppb. The measurement lasted for 12-48 hours and was conducted three times. No DO was detected.

6. Quantification of ATP in suspension and slurry samples

The suspension with copper compounds and cells as mentioned in *section 4* was analyzed by the QG21W-50C kit designed for wastewater and suspended growth systems. For ATP analysis, we followed the instructions from the manufacturer. Briefly, after checking the enzyme with the UltraCheck solution, the total ATP (tATP) in the liquid was measured (need ~2 min) and then the dissolved ATP (dATP) was measured (need ~2 min). The RLU values from the PhotonMaster luminometer recorded during the above measurements were used for calculations. Specifically, the total, dissolved, and intracellular ATP (cATP) were computed following these equations.

a) tATP

$$tATP(ng\ \mathrm{ATP}/mL) = \frac{RLU_{tATP}}{RLU_{ATP1}} \times 11(ng\ \mathrm{ATP}/mL)$$

b) dATP

$$dATP(ng\ \mathrm{ATP}/mL) = \frac{RLU_{dATP}}{RLU_{ATP1}} \times 101(ng\ \mathrm{ATP}/mL)$$

c) cATP

$$cATP\,(ng\,ATP/\,mL) = tATP - dATP\,(ng\,ATP/\,mL)$$

After obtaining the cATP, the AVSS can be calculated by the equation below.

$$AVSS\,(mg\,Biomass/\,L) = cATP\,(ng\,ATP/\,mL) * 0.5$$

A second ATP kit from LuminUltra Technologies Ltd., the DSA kit (Deposit & Surface

Analysis), was used for quantifying ATP in slurry samples. Specifically, the reagents were all moved to the bench top and allowed to warm until they reached room temperature. The rehydrated Luminase was calibrated every time before actual analysis with the UltraCheck1 solution according to the manufacturer's instructions. Immediately insert the tube holding the mixture into the luminometer and measure. Record RLU_{ATP1}. Obtain a portion of the slurry after opening the glass bottles and weigh 1g of the sample. Add this to a 5mL UltraLyse 7 Tube. Cap and mix the contents of the tube vigorously to disperse the deposit throughout the fluid. The slurry was incubated in the UltraLyse tube for 5 min. After the incubation, 1 ml of the extraction solution was transferred to the UltraLute tube, and cap and invert the tube three times for mixing. Transfer 100μL from the UltraLute Tube to a new test tube and mix with 100μL of Luminase, swirl gently five times, and then insert the tube into the luminometer for reading, record as RLU_{tATP}. The total ATP (tATP) was calculated according to the equation provided by the manufacturer, as below.

$$tATP(pg\ ATP/g) = \frac{RLU_{tATP}}{RLU_{ATP1}} \times \frac{50000(pg\ ATP)}{m_{sample}\ (g)}$$

According to the manufacturer, to communicate results on the same basis as traditional culture tests, tATP results can be converted into Microbial Equivalents (ME's). This is based on the established conversion that 1 E. coli-sized bacterial cell contains 0.001 pg of ATP.

$$tATP(ME/g) = tATP(pg\ ATP/g) \times \frac{1\ ME}{0.001\ pg\ ATP}$$

7. Metagenome sequencing, and bioinformatics analyses

For meta-genomic sequencing, the raw data were trimmed using Trimmomatic to remove low-quality sequences, library primers and adapters and quality-filtered using fastq_quality_filter from the FASTX toolkit with default settings. Filtered reads were assembled into contigs with MEGAHIT (Version 1.2.9, https://github.com/voutcn/megahit). For metagenome assembly, MEGAHIT [7] was used and the k-mer values for de novo assembly are k-min of 35, k-max of 95, and k-step of 20. To use the information obtained by scaffolding, Scaftigs were constructed by extracting the contiguous sequences that lack unknown bases (Ns). Only those larger than 500 bp were used for subsequent analysis. The clean data from each sample were further compared to the respective scaffolds to identify the reads not used, which were used for mixed assembly with MEGAHIT for low-abundance species in the samples.

Protein-coding regions or ORF were predicted from the Scaftigs (≥500 bp) with MetaGeneMark (Version 3.38): http://exon.gatech.edu/GeneMark/metagenome/Prediction, and those sequence lengths shorter than 90 nt were filtered from the predicted results with default parameters. The CD-HIT (Version: 4.7): http://www.bioinformatics.org/cd-hit/ was used

to remove redundancy and obtain the unique initial gene catalog (unigenes), clustered at 95% identity with 90% coverage of the longest representative sequences. The clean data of each sample was mapped to the initial gene catalog using BBMAP software (http://jgi.doe.gov/data-and-tools/bbtools/) to get the number of reads that can be mapped to genes in each sample. The relative abundance of each gene was then computed according to the number of mapped reads and the length of genes [8]. The basic statistics, core-/pan-genome analysis, and correlation analysis were all based on the abundance of each gene in the respective sample. DIAMOND software (https://github.com/bbuchfink/diamond/) was used to conduct BLAST analysis of the non-redundant unigenes against the sequences of Bacteria, Fungi, Archaea, and Viruses from the NR database of NCBI (those data related to Fungi and Viruses were not shown in the paper). LCA (lowest common ancestor) algorithm in the MEGAN package was used for binning reads onto the nodes of the NCBI taxonomy based on alignments ($e=10^{-10}$). The table containing the avg depth of genes and the abundance information of each sample in each taxonomy hierarchy (kingdom, phylum, class, order, family, genus, species) were obtained based on the LCA annotation results and the gene avg depth table or the gene abundance table. The abundance of a species in one sample equals the sum of the abundance of all genes annotated to the species; similarly, the gene avg depth of a species equals the sum of all the corresponding nonzero gene avg depth. Cluster analysis and PCoA (Principal Coordinates Analysis, based on Bray–Curtis distance metrics) were based on the abundance table of each taxonomic hierarchy. The unweighted pair group method with arithmetic mean (UPGMA) clustering was applied as a type of hierarchical clustering method to interpret the distance matrix using QIIME software (Version 1.7.0). Furthermore, DIAMOND was used to align the unigene sequences against the protein reference sequences from KEGG database (http://www.kegg.jp/kegg/) [9].

8. Additional figures and table

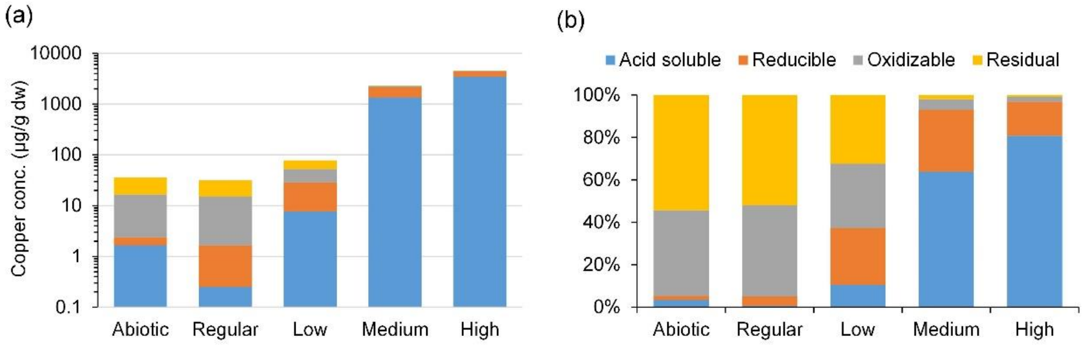

Figure S1. Cu levels and speciation after the test with $CuCl_2$ on the DAMO enrichment. (a) and (b) shows the sequential extraction results in absolute mass concentrations and proportions, respectively.

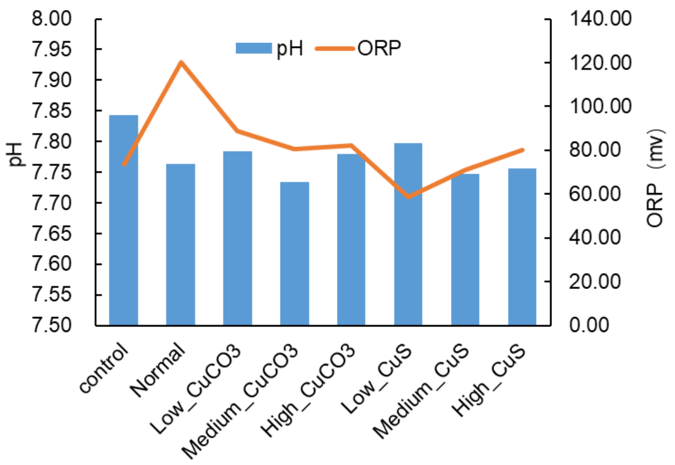

Figure S2. The pH and ORP measured after the 54-day toxicity tests with copper.

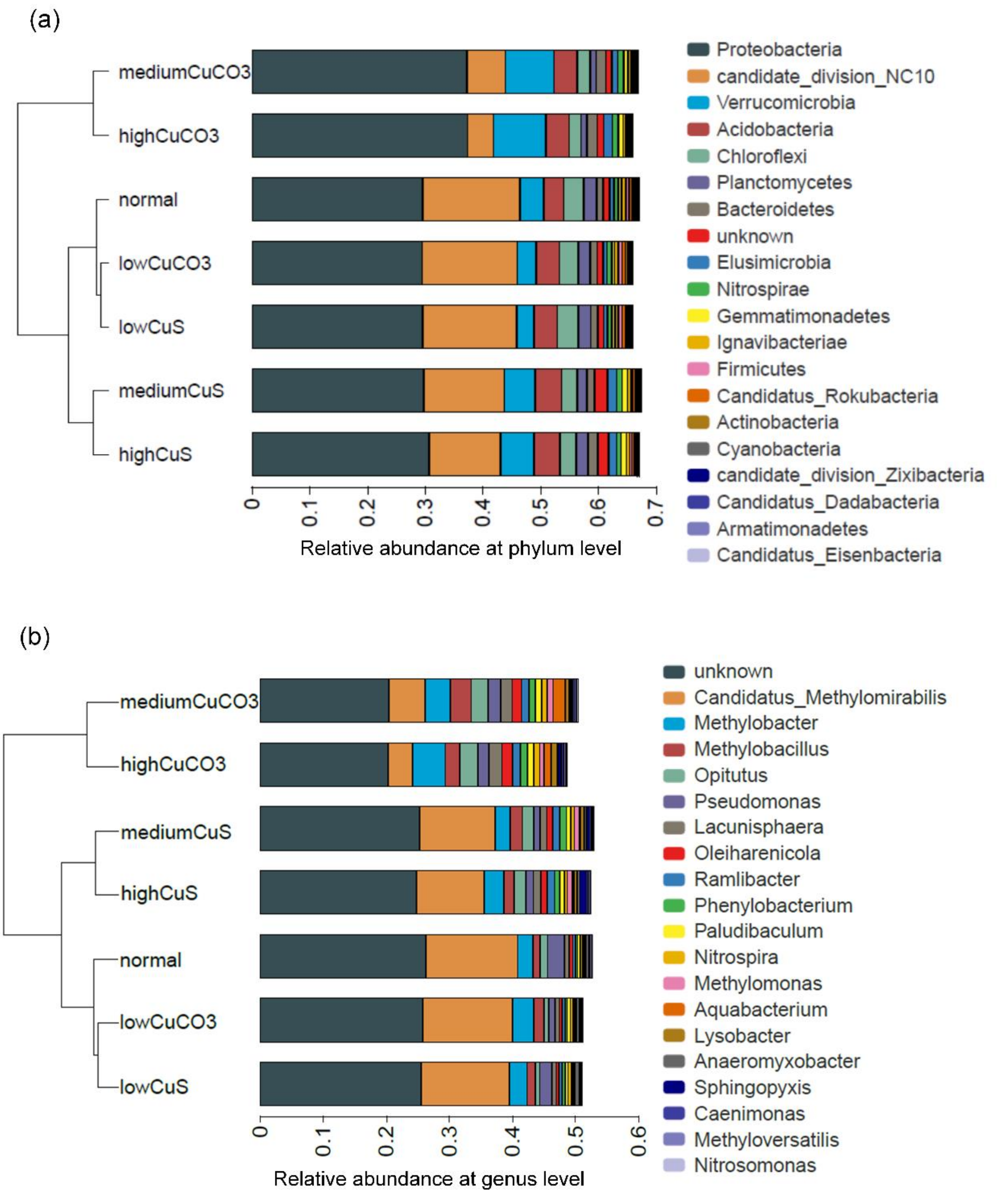

Figure S3. Cluster analysis of the control and test groups with hierarchical clustering analysis by UPGMA on the left and relative abundance at the (a) phylum and (b) genus level on the side (top 20 most abundant).

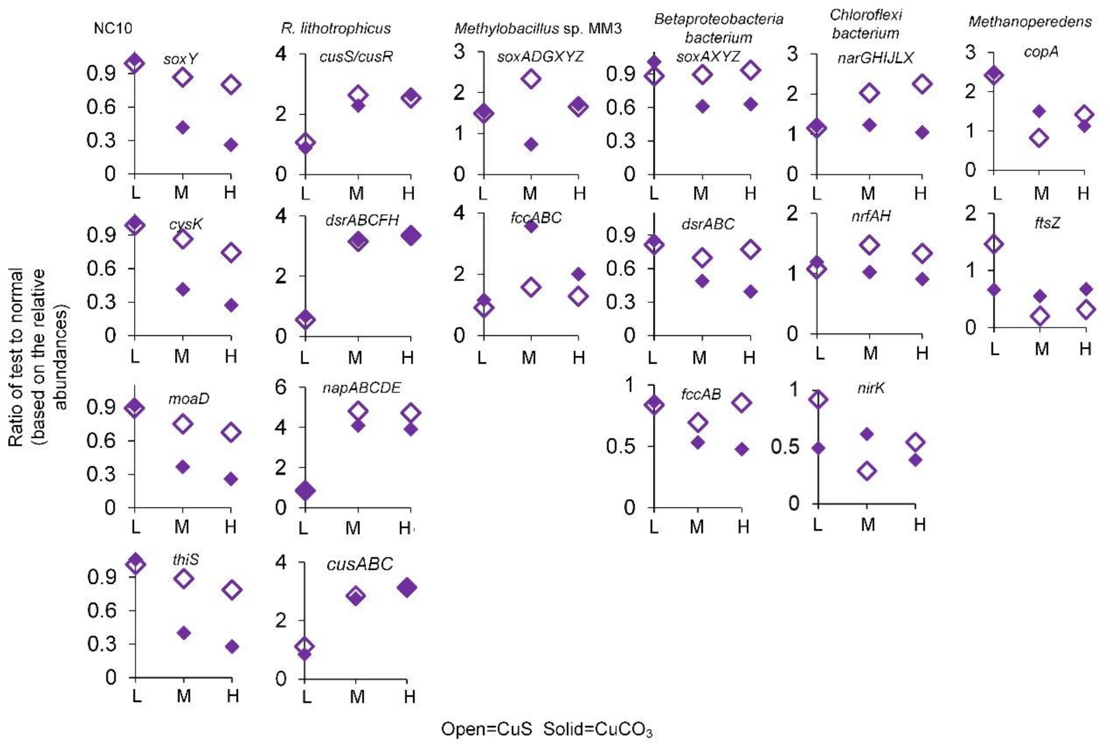

Figure S4. Variation of representative genes as compared to the normally cultivated group from major taxa.

Table S1. qPCR primers and PCR protocols used in this study.

Microorganism	Target gene	Primers	Sequence (5'-3')	PCR conditions	Reference
DAMO bacteria	*pmoA*	cmo182	TCACGTTGACGCCGATCC	94℃ for 4min, 35×[94℃ for 1min, gradient(50℃-60℃) for 1min, 72℃ for 1min], 72℃ for 10min	Luesken et al., 2011
		cmo568	GCACATACTCCATCCCCATC		
DAMO archaea	*mcrA*	McrA159F	AAAGTGCGGAGCAGCAATCACC	96 °C for 5 min, 45×[96 °C for 30 s, gradient (55–68 °C) for 45 s, and 72 °C for 45 s], 72 °C for 10 min	Vaksmaa et al., 2017
		McrA345R	TCGTCCCATTCCTGCTGCATTGC		
Total bacteria	*16S rRNA*	341F	CCTACGGGAGGCAGCAG	50 °C for 2 min, 94 °C for 30 s, 35 × [95 °C for 5 s, 58 °C for 15 s, 72 °C for 35 s], 72 °C for 5 min; melt curve 60-95° C C	Shen et al., 2019
		518R	ATTACCGCGGCTGCTGG		
Total archaea	*16S rRNA*	Arch967F	AATTGGCGGGGGAGCAC	50 °C for 2 min, 94 °C for 10 min, 38 × [95 °C for 15 s, 60 °C for 1 min], 72 °C for 5 min; melt curve 60-95° C	Shen et al., 2019
		Arch1060R	GGCCATGCACCWCCTCTC		

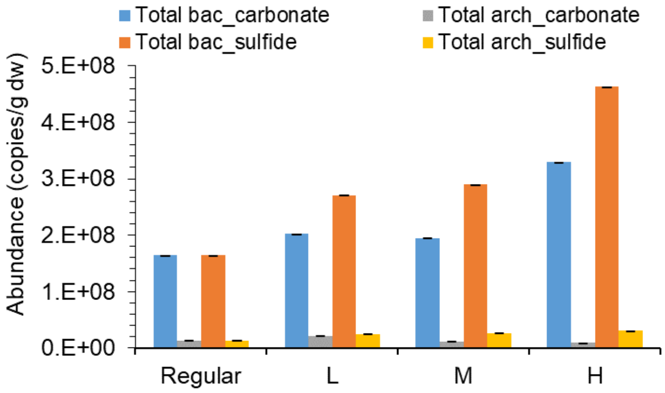

Figure S5. Absolute gene copies of general bacteria and archaea in the tested groups after 54 days.

9. References:

1. Gao, Y.; Trueman, B.F.; Li, B.; Earle, M.R.; Gagnon, G.A. Release and migration of Pb from Pb(ii) and Pb(iv) compounds in the presence of microbiological activity. *Environmental Science: Water Research & Technology* **2022**, *8*, 2905-2916, doi:10.1039/D2EW00152G.
2. Gao, Y.; Trueman, B.F.; Gagnon, G.A. Early phase effects of silicate and orthophosphate on lead (Pb) corrosion scale development and Pb release. *Journal of Environmental Management* **2022**, *321*, 115947, doi:https://doi.org/10.1016/j.jenvman.2022.115947.
3. Ure, A.M.; Quevauviller, P.; Muntau, H.; Griepink, B. Speciation of Heavy Metals in Soils and Sediments. An Account of the Improvement and Harmonization of Extraction Techniques Undertaken Under the Auspices of the BCR of the Commission of the European Communities. *International Journal of Environmental Analytical Chemistry* **1993**, *51*, 135-151, doi:10.1080/03067319308027619.
4. Hu, S.; Hu, J.; Sun, Y.; Zhu, Q.; Wu, L.; Liu, B.; Xiao, K.; Liang, S.; Yang, J.; Hou, H. Simultaneous heavy metal removal and sludge deep dewatering with Fe(II) assisted electrooxidation technology. *Journal of Hazardous Materials* **2021**, *405*, 124072, doi:https://doi.org/10.1016/j.jhazmat.2020.124072.
5. Rao, C.R.M.; Sahuquillo, A.; Lopez Sanchez, J.F. A Review of the Different Methods Applied in Environmental Geochemistry For Single and Sequential Extraction of Trace Elements in Soils and Related Materials. *Water, Air, and Soil Pollution* **2008**, *189*, 291-333, doi:10.1007/s11270-007-9564-0.
6. Gao, Y.; Ryu, H.; Rittmann, B.E.; Hussain, A.; Lee, H.-S. Quantification of the methane concentration using anaerobic oxidation of methane coupled to extracellular electron transfer. *Bioresource Technology* **2017**, *241*, 979-984, doi:https://doi.org/10.1016/j.biortech.2017.06.053.
7. Li, D.; Liu, C.-M.; Luo, R.; Sadakane, K.; Lam, T.-W. MEGAHIT: an ultra-fast single-node solution for large and complex metagenomics assembly via succinct de Bruijn graph. *Bioinformatics* **2015**, *31*, 1674-1676, doi:10.1093/bioinformatics/btv033.
8. Qin, J.; Li, Y.; Cai, Z.; Li, S.; Zhu, J.; Zhang, F.; Liang, S.; Zhang, W.; Guan, Y.; Shen, D.; et al. A metagenome-wide association study of gut microbiota in type 2 diabetes. *Nature* **2012**, *490*, 55-60, doi:10.1038/nature11450.
9. Buchfink, B.; Xie, C.; Huson, D.H. Fast and sensitive protein alignment using DIAMOND. *Nature Methods* **2015**, *12*, 59-60, doi:10.1038/nmeth.3176.
